# Effects of home-based manual dexterity training on cognitive function among older adults: a randomized controlled trial

**DOI:** 10.1186/s11556-023-00319-2

**Published:** 2023-04-22

**Authors:** Jaehoon Seol, Namhoon Lim, Koki Nagata, Tomohiro Okura

**Affiliations:** 1 Research Center for Overwork-Related Disorders, National Institute of Occupational Safety and Health, Japan (JNIOSH), Kawasaki, Japan; 2 International Institute for Integrative Sleep Medicine (WPI-IIIS), University of Tsukuba, Tsukuba, Japan; 3 R&D Center for Tailor-Made QOL, University of Tsukuba, 1-2 Kasuga, Tsukuba, Ibaraki 305-8550 Japan; 4 Doctoral Program in Physical Education, Health and Sports Sciences, Graduate School of Comprehensive Human Sciences, University of Tsukuba, Tsukuba, Japan; 5 Faculty of Health and Sport Sciences, University of Tsukuba, Tsukuba, Japan

**Keywords:** Executive function, Stroop Color and Word Test, Purdue Pegboard Test, Peg-Amore, Digit training

## Abstract

**Background:**

The relationship between manual dexterity and cognitive function among older adults is well known; however, few studies have focused on manual dexterity training that confirms cognitive load of training in older adults through functional near-infrared spectroscopy (fNIRS) and verifies the effect of training. This study examined the effects of home-based manual dexterity training on cognitive function in older adults using a digital trail-making peg test device combining two conventional assessment tools namely, the peg and trail-making tests.

**Methods:**

For 12 weeks, 57 healthy older adults aged 65–88 years participated in a parallel-group, randomized controlled trial, wherein home-based manual dexterity training was performed for approximately 20 min daily. To quantify the cognitive load in different manual dexterity conditions, we assessed the cortical activation patterns of the prefrontal cortex via a wearable four-channel fNIRS device. Participants in the control group were asked to continue their usual daily routines during the intervention period. Cognitive function was assessed using the Stroop Color and Word and Cognitive Impairment Tests. Manual dexterity was assessed using the Purdue Pegboard Test. All outcomes were estimated before and after the intervention.

**Results:**

We observed significant differences in prefrontal cortical activation between the different manual dexterity conditions. Only the intervention group showed a significant improvement in Stroop interference (169.0–108.9 ms, *p* = 0.032) and an executive function and assembly task of the Purdue Pegboard Test (22.5–26.4 counts, *p* < 0.001). Additionally, except the clock drawing task, cognitive function had a larger effect size (Cohen’s *d*) in the intervention group (*d* = 0.26–0.45) than in the control group (*d* = 0.11–0.28).

**Conclusions:**

Home-based manual dexterity training can improve performance in a complex manual dexterity task and executive functioning in older adults.

**Trial registration:**

UMIN-CTR Clinical Trial, UMIN000047203. Registered 17 March 2022 – Retrospectively registered, https://center6.umin.ac.jp/cgi-open-bin/ctr/ctr.cgi?function=brows&action=brows&recptno=R000053844&type=summary&language=E

**Supplementary Information:**

The online version contains supplementary material available at 10.1186/s11556-023-00319-2.

## Background

Among the many deleterious effects of aging, deterioration of manual dexterity is one that can lead to difficulty in performing instrumental activities of daily living, such as writing, cooking, gardening, working on crafts, and opening a bottle [1–3]. These principal aging-associated changes are observed in both men and women, especially those older than 65 years [4–6]. Preserving manual dexterity is essential for daily living in older adults [2].

Previous studies have revealed that manual dexterity is positively related to cognitive functions, particularly the executive function, which includes inhibitory control, working memory, and cognitive flexibility [2, 4, 7, 8]. Manual dexterity is related to not only executive function but also muscle strength [2, 9]. Moreover, the performance of executive functions is related to the motor control performance (i.e., manual dexterity) of both hands, especially the non-dominant hand in older adults [3, 10, 11]. Manual dexterity is particularly important to ensure independent living of older adults owing to its association with the performance of instrumental activities of daily living [6].

Performing tasks that require manual dexterity activates the prefrontal cortex, primary motor cortex, and primary sensorimotor cortex in older adults [1, 12, 13]. The primary motor cortex and corticomotoneuronal cells that synapse directly onto spinal motor neurons, bypassing the spinal interneurons, are involved in manual dexterity; hand movement with visual information involves the primary motor cortex and associated neurons [1]. This corticomotoneuronal pathway is critical for individual finger movements and for grasping behaviors in primates, including humans [1]. Corresponding to this, a cross-sectional epidemiologic study indicated that hand use performance assessments, such as handgrip strength [9] and manual dexterity [2], are useful tools not only in estimating the status of cognitive function but also in screening for mild cognitive impairment status in early stages [10, 14, 15].

Additionally, manual dexterity has a training effect in older adults [6, 16]. Our previous experimental study showed that participants who performed an acute bout of manual dexterity movement had higher executive function than sedentary controls, and the effect size was greater than that of step exercises and stretching performed while sitting [17]. Another intervention study lasting 2 months reported the effects of manual dexterity training (i.e., involved finger extension, counting, rotation, rock-paper-scissors, and shape generation) on manual dexterity and cognitive function. Compared to the only dominant training group, the training group using both hands improved manual dexterity and demonstrated a more pronounced activation of the primary motor cortex during a pegging task [12]. Based on previous studies [12, 17], it seems reasonable to hypothesize that manual dexterity training could have a positive effect on manual dexterity and executive function in older adults. Interestingly, an acute experimental study revealed that compared to the easier task (control, and trail-making test (TMT) part A), the difficult task (TMT part B) shows greater activation of the prefrontal cortex (PFC) in younger adults [18]. However, only a few intervention studies exist that confirmed the cognitive load of training in older adults through fNIRS and verified the effect of training [3].

Based on the evidence provided by the abovementioned studies (e.g., [18]), we hypothesize that the PFC can be stimulated by manual dexterity training [19], and thus, the 12-weeks training would improve manual dexterity and cognitive function in older adults.

## Methods

### Study design

A parallel-group, randomized controlled trial was conducted from September 2021 to February 2022 (Supplementary Fig. S1). PFC activation of (i.e., cognitive load) was confirmed during the three-mode (P-, A-, and B-modes) digital trail-making peg test using fNIRS during baseline measurement before randomization (Supplementary Fig. S2). After randomization, manual dexterity and cognitive function tests were performed by all participants before and after the intervention (Supplementary Fig. S1), and the results were recorded.

### Participants

All participants were recruited using a local newspaper that was distributed throughout Ibaraki Prefecture, Japan. Initially, 164 older adults aged 65–88 years volunteered for the study. We included adults aged > 65 years who were right-handed, had no color blindness, had no physician-imposed exercise restrictions, and were not involved in any experimental research during the past year. Of the remaining 73 people who were eligible, 60 were randomly selected by a blinded third researcher using Excel version 1902. After intervention, individuals who were ambidextrous (*n* = 1) or had considerable cognitive impairment based on a Mini-Mental State Examination score of ≤ 26 (*n* = 2) were excluded from the analysis (Supplementary Fig. S1) [20]. All participants provided signed informed consent and no monetary compensation was provided. All protocols were approved by the ethics committee of the University of Tsukuba (Ref. Tai 021–34; Tai 021–42). The study protocol (UMIN000047203) was retrospectively registered with the University Hospital Medical Information Network Center (http://www.umin.ac.jp/english/).

### Sample size

The optimal sample size was determined using G*Power version 3.1 based on a 0.25-point effect size, α level of *p* < 0.05, and 95% power as analysis of variance (ANOVA) designs [21]. The results indicated that 24 participants were required for each group. Assuming a 15% dropout rate, the minimum sample size was determined to be 56 participants.

### Randomization

Baseline measurements were collected. After stratification by sex (female, *n* = 43; male, *n* = 17) [22], participants were randomized into either the intervention group (*n* = 30) or control group (*n* = 30) using the Excel version 1902 randomizer function.

### Interventions

The intervention consisted of a 12-week training program that included seven different elements with the aid of a training device that recorded the results. We developed a digital trail-making peg test device (Peg-Amore [41 cm × 35 cm × 5 cm]; NEWCOM Inc., Saitama, Japan) (Supplementary Fig. S3). This training device, which combines the elements of both the twenty-five-hole peg test [23] and TMT [24], was used to evaluate manual dexterity and cognitive status in occupational therapy areas in Japan (Japan Patent 2019–024,707) [25]. The device was provided to the intervention group.

The participants were taught how to conduct the training and record the results. The training schedule required the participants to exercise their right hand on Monday, Wednesday, and Friday and their left hand on Tuesday, Thursday, and Saturday; the non-dominant hand had to be trained on Sunday. The daily training regimen would take approximately 20 min daily. The training program consisted of seven modes that were intended to stimulate elements of executive, attention, memory, and dynamic vision functions, as described in Supplementary Methods. After a familiarization period, the participants completed their dexterity training for each mode.

The digital trail-making peg test device was able to record and store the participants’ data. After the intervention period, all of the device’s data were cross-checked with the participants’ self-reported training diary and the adherence rate calculated. The training device had a “guest” mode to distinguish the participants’ data from that of a family member, friend, or guest who may have used the device. The intervention group received personal feedback and encouragement from the researcher every week via telephone.

The control group was asked to perform their daily activities as usual during the intervention period. For ethical reasons, the control group received the same training program after the intervention period was over.

Among all training modes, the A- and B-modes were based on TMT-A and TMT-B (Supplementary Methods). TMT is a widely used test of executive function, and the calculated B-A time and B/A ratio are related to working memory, processing speed, and general cognitive function [26, 27]. Our previous study confirmed the validity and reliability of general cognitive function among older adults in the A- and B-modes [28]. Thus, we adopted the B-A time and B/A ratio using A-mode (trail-making peg test part A) and B-mode (trail-making peg test part B) during the intervention period.

## Measurements

### fNIRS data acquisition and analysis

To confirm the cognitive load of the manual dexterity training, blood oxyhemoglobin, deoxyhemoglobin, and total hemoglobin levels were measured using fNIRS. We used the Hb-131S fNIRS device (Astem Corp., Fukuoka, Japan) with four active channels (two colors of LEDs, λ_1|2_: 770|830 nm with average power 1 mW or less, and four avalanche photodiode light detectors) sampled at 10 Hz. Data were converted to concentration changes using the modified Beer–Lambert law [29, 30]. Fpz in the 10–20 electrode system corresponds to the center of the fNIRS cap [31] to estimate the PFC. Moreover, we removed the movement artifact using the three axes accelerometer data attached to the fNIRS and algorithms described previously [32]. The concrete position and details of the device have been described previously [33].

The participants’ cognitive load changes were measured during the P-mode (simple peg moving training), A-mode (the participants grasp 25 pegs, one at a time with one hand, and place them in vertically aligned holes as rapidly as possible in the numerical order displayed on the peg board [i.e., 1 → 2 → 3, …, 24 → 25]), and, after at least 5 s of rest, B-mode (i.e., using a combined number and Japanese characters Hiragana order, the participants grasp a peg and alternately move it to the correct hole in the displayed order [1 → [a] → 2 → [i] → 3 → [u], …, [shi] → 13]). In this study, we used a block design with a 5-s-long baseline period and 25 sub-trials of each test (P-mode, A-mode, and B-mode). The rest interval between the task blocks was longer than 30s.

Owing to technical problems (i.e., data not calculated for unknown reason), data were missing for four participants of fNIRS data; 37 channels of cumulative 636 channels were omitted due to bad signal quality. Overall, the data of 53 fNIRS procedures were analyzed. The fNIRS data were registered to the Montreal Neurological Institute space, and the channel location was measured using the three-dimensional spatial position of the channels measured by a digitizer (FASTRAK, Polhemus Inc., Colchester, VT, USA).

Data processing was performed using the Open Platform of Transparent Analysis Tools for fNIRS (Open PoTATo) for MATLAB R2020a. All channels were preprocessed with a low-pass filter using a cut-off frequency of 0.2 Hz based on a similar previous study that measured fNIRS during the TMT [18]. We used a 5-s set zero-level function for Open PoTATo. The calculated oxy-Hb levels for each task are shown in Supplementary Fig. S2. The actual elapsed times varied among the participants; thus, we separated the average baseline oxy-Hb (5 s) and average of each oxy-Hb quartile (25%, 50%, 75%, and 100%). All the Oxy-Hb and deoxy-Hb data are presented in Supplementary Table S1.

### Cognitive functions

Executive function was assessed using the Stroop Color and Word Test consisting of neutral, congruent, and incongruent trials [34]. Two rows of messages appeared on the computer screen. The bottom row consistently contained the words YELLOW, GREEN, RED, or BLUE. In the neutral trial, the top row, displayed “XXXX” in one of four colors: red, blue, green, or yellow. The bottom row words were printed in black. In the congruent trial, the top row contained the words RED, GREEN, BLUE, or YELLOW printed in a color congruent with the words in the bottom row. In the incongruent trial, the top row contained the words YELLOW, GREEN, RED or BLUE in a color incongruent with the text color of the bottom row words. In all trials, participants were asked whether the name of the color that appeared in the bottom row was the same as the color of what appeared in the top row. Each session consisted of 30 trials, including 10 neutral, 10 congruent, and 10 incongruent trials in random order [35]. The average reaction time was used for analysis. Stroop interference was calculated as the difference between the reaction times of incongruent and congruent tasks [34, 36].

Memory and judgment function were assessed using the Cognitive Impairment Test, which is also one of the tests required for driver license renewal among older adults in Japan [37]. We adopted this cognitive test since it was easy to generalize the changes in the scores of Japanese older adults. Details of the test and scoring have been described previously [38]. This test consists of three tasks: orientation to time (0–15 points), free and cued recall (0–32 points), and clock drawing (0–7 points). The total score is calculated using the following equation:

$$\mathrm{Total}\;\mathrm{score}\;=\;(1.15\times\mathrm{orientation\;to\;time})+(1.94\times\mathrm{free\;and\;cued\;recall})+(2.97\times\mathrm{clock\;drawing})$$

### Manualdexterity

The Purdue Pegboard Test (model 32,020; Lafayette Instrument Co., Lafayette, IN, USA) was used to assess manual dexterity. The Purdue Pegboard Test is widely used in both clinical and research fields, and its methods, validity and reliability have been confirmed [39]. The number of pegs successfully pinned on the pegboard in three trials lasting 30 s using the left, right, and both hands was counted. The assembly task involved assembling pins, washers, and collars alternately using both hands for 60 s.

### Potential confounding factors

Demographic variables included age, sex, body mass index (kg/m^2^), medication history, smoking status (current or past/never), drinking habits (daily/1–6 times per week/ < 1–3 times per month), years of education, and depressive symptoms (15-item Geriatric Depression Scale) [40]. Daily physical activity level, social relationships, and sleep quality were assessed using the Physical Activity Scale for the Elderly [41], Lubben Social Network Scale [42], and Pittsburgh Sleep Quality Index [43], respectively.

### Statistical analysis

We intended to perform ANOVA; however, owing to the presence of missing data (i.e., 37 channel data of cumulative 636 channels data were omitted due to bad signal quality), we analyzed the fNIRS signals during the P-, A-, and B-modes using mixed-model ANOVA instead. Group differences at baseline were determined using unpaired *t*-tests and chi-square tests for continuous and categorical variables, respectively. Two-way repeated-measures ANOVA was performed to evaluate the differences in the effect between groups (intervention vs. control) and time (pre-test vs. post-test), and a post hoc test was conducted on variables showing significant group-by-time interaction. Missing data (eight participants dropped out) were filled in with baseline observations from the exit date using intention-to-treat analysis [44]. We confirmed the normal distribution of data using the Shapiro–Wilk test; however, certain outcomes (Purdue Pegboard Test and Cognitive Impairment Test) were not normally distributed. The average value for each week was analyzed using mixed-model ANOVA based on the recorded log-data of manual dexterity training tasks during the intervention period. Bonferroni post hoc tests were used to correct for multiple comparisons.

Pearson’s correlation coefficients were calculated using the changes in the primary outcomes (Stroop task, Cognitive Impairment Test, and Purdue Pegboard Test) and executive function (B-A time and B/A ratio). Specifically, change in the primary outcomes was defined as the amount of change from post-intervention to pre-intervention, as determined by subtracting the post-intervention results from the pre-intervention results. The change in the executive function performance using the digital trail-making peg test device was defined as the difference between the average score obtained during the 12th-week and the average score obtained during the 1st-week period (a negative number indicated functional improvement). The correlation coefficient was classified as no correlation (*r* = 0–0.19), low correlation (*r* = 0.20–0.39), moderate correlation (*r* = 0.40–0.59), moderate high correlation (*r* = 0.60–0.79), and high correlation (*r* ≥ 0.80) [45]. Effect size was determined using Cohen’s *d* [46]. The effect size was classified as small (*d* = 0.2), medium (*d* = 0.5), and large (*d* = 0.8) [46].

Statistical significance was set at *p* < 0.05 (two-tailed). All analyses were performed using SPSS for Windows version 28.0 (IBM Corp., Armonk, NY, USA).

## Results

### Participant characteristics

Baseline participant characteristics are presented in Table 1. Among 57 participants, 49 completed the study (dropout rate, 14.1%). Attrition was due to personal and environmental reasons (hospitalization [*n* = 1], coronavirus disease [*n* = 1], and fear of contracting coronavirus disease [*n* = 6]); all factors were unrelated to the study (Supplementary Fig. S1). The completion rate of this training calculated based on log-data was 88.7 ± 13.9% in the intervention group. Supplementary Figs. S4 and S5 show the improvement in each training mode during the intervention period (all *p* values for trend were < 0.001).

**Table 1:** Participants’ characteristics at baseline

	Total participants (*n* = 57)			Intervention group (*n* = 28)			Control group (*n* = 29)			Unpaired *t* test or chi-square*p* value
	Mean	±	SD	Mean	±	SD	Mean	±	SD	
Age, years	73.6	±	6.1	72.9	±	5.6	74.4	±	6.5	0.348
Female, n (%)	39	(68.4)		19	(67.9)		20	(69.0)		0.928
Body mass index, kg/m^2^	22.8	±	3.6	23.1	±	3.1	22.5	±	4.0	0.503
Smoking habit, n (%)	1	(1.8)		1	(3.6)		0			0.305
Alcohol consumption (drinker), n (%)	20	(35.1)		12	(42.9)		8	(27.6)		0.227
Educational level, years	14.1	±	2.8	13.9	±	2.3	14.6	±	2.0	0.203
PASE score, points	125.9	±	67.7	141.9	±	85.8	110.5	±	39.6	0.085
MMSE score, points	29.6	±	0.7	29.6	±	0.7	29.6	±	0.7	0.801
**Medical history**										
None, n (%)	26	(45.6)		14	(50)		12	(41.4)		0.514
Hypertension, n (%)	16	(23.5)		9	(32.1)		7	(24.1)		0.501
Hyperlipidemia, n (%)	10	(17.5)		5	(17.9)		5	(17.9)		0.951
Diabetes, n (%)	5	(8.8)		2	(7.1)		3	(10.3)		0.669
**LSNS score, points**	16.8	±	5.4	17.2	±	6.0	16.3	±	4.7	0.563
Social isolation (< 12 points), n (%)	10	(17.5)		5	(17.9)		5	(17.2)		0.951
**PSQI score, points**	5.0	±	2.4	4.9	±	2.6	5.1	±	2.3	0.829
Poor sleeper (> 5.5 points), n (%)	21	(36.8)		9	(32.1)		12	(41.4)		0.470
**GDS score, points**	3.6	±	2.6	3.7	±	2.5	3.5	±	2.6	0.814
Depression (> 5 points), n (%)	18	(31.6)		9	(32.1)		9	(31.0)		0.928

*SD* Standard deviation, *PASE*, Physical Activity Scale for the Elderly, *MMSE* Mini-Mental State Examination, *LSNS* Lubben Social Network Scale, *PSQI* Pittsburgh Sleep Quality Index, *GDS* Geriatric Depression Scale 15

### Assessment of fNIRS cognitive load data

**Fig. 1:**
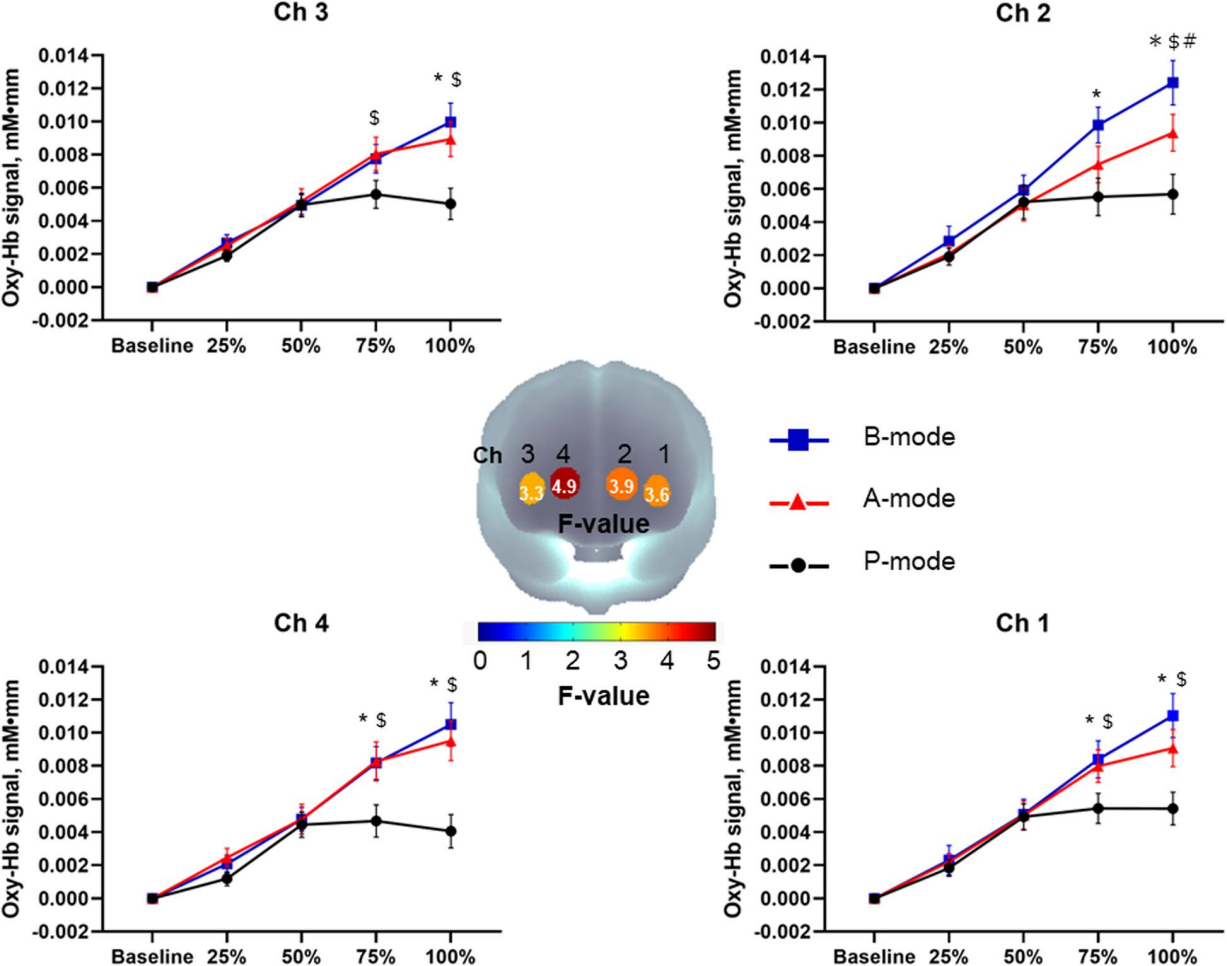
Distinct effect of frontal lobe activation during each mode. F-values are denoted according to the color bar. Y-axis unit indicates oxy-Hb signal (mM · mm). Error bars indicate standard error. $, significant differences between P- and A-modes; *, significant differences between P- and B-modes; # significant differences between A- and B-modes

The oxy-Hb concentration of all channels during tasks showed significant effects of time (*p* < 0.001), group (*p* < 0.001), and interaction (*p* < 0.001) (Supplementary Table S1, and Fig. 1). Compared to the P-mode, the A- and B-modes showed a significant increase after 50–100%, and the post hoc analysis revealed that the A- and B-modes were significantly higher than the P-mode at 75% and 100% points in channels 1, 3, and 4. Channel 2 showed that only the B-mode was significantly higher than the P-mode at the 75% point, and all modes were significantly different at the 100% point (all *p* < 0.05) (Supplementary Table S1, and Fig. 1).

### Cognitive function

Stroop interference showed significant effects of interaction (*p* = 0.025). The post hoc test showed a significant improvement after training in the intervention group (169.0 ± 109.2 to 108.9 ± 110.9 ms; *p* = 0.032). The reaction time was not significantly different between the neutral, congruent, and incongruent tasks; however, the effect size of the neutral (*d* = 0.22 and 0.13) and incongruent tasks (*d* = 0.46 and 0.19) was higher in the intervention group than in the control group (Table 2). The Cognitive Impairment Test did not show significant differences; however, the effect size of total score in the intervention group was higher than that in the control group (*d* = 0.27 and 0.12) (Table 2).

**Table 2:** Change in executive function, cognitive function, and manual dexterity during the intervention period

		Intervention group (*n* = 28)				Control group (*n* = 29)				Interaction *p*	Group effect *p*	Time effect *p*
		Mean	±	SD	Effect size Cohen's *d*	Mean	±	SD	Effect size Cohen's *d*			
**Stroop Color and Word Test, ms**												
Neutral task	Pre	1176.7	±	153.7	0.22	1192.0	±	168.6	0.13	0.630	0.596	0.067
	Post	1138.6	±	192.6		1169.6	±	176.4				
Congruent task	Pre	1199.9	±	138.2	0.16	1247.7	±	169.2	0.33	0.229	0.360	0.031
	Post	1175.6	±	159.2		1191.9	±	168.0				
Incongruent task	Pre	1368.9	±	169.8	0.46	1350.1	±	155.2	0.19	0.349	0.418	0.020
	Post	1284.6	±	196.6		1321.9	±	136.5				
Stroop interference task	Pre	169.0	±	109.2	0.55	102.4	±	138.6	-0.19	0.025	0.395	0.395
	Post	108.9	±	110.9*		129.9	±	155.2				
**Cognitive Impairment Test, points**												
Orientation to time	Pre	14.6	±	0.9	0.45	14.8	±	0.4	0.28	0.224	0.387	0.123
	Post	14.9	±	0.3		14.9	±	0.3				
Free and cued recall	Pre	24.4	±	5.4	0.26	24.9	±	5.4	0.11	0.393	0.910	0.034
	Post	25.8	±	5.3		25.5	±	5.2				
Clock drawing	Pre	6.9	±	0.3	< 0.01	6.8	±	0.5	< 0.01	0.423	0.739	0.989
	Post	6.9	±	0.4		6.8	±	0.4				
Total score	Pre	84.6	±	11.1	0.27	85.7	±	11.1	0.12	0.379	0.908	0.024
	Post	87.5	±	10.7		87.0	±	10.2				
**Purdue Pegboard Test, count**												
Right hand task	Pre	12.4	±	1.9	0.32	12.0	±	2.1	0.22	0.746	0.390	0.006
	Post	13.0	±	1.9		12.5	±	2.4				
Left hand task	Pre	12.5	±	1.4	0.36	11.8	±	1.9	0.25	0.966	0.087	0.018
	Post	13.0	±	1.4		12.3	±	2.1				
Both hand task	Pre	9.6	±	1.4	0.57	9.5	±	1.9	0.36	0.944	0.515	< 0.001
	Post	10.4	±	1.4		10.1	±	1.4				
Sum of right-, left-, and both hand task	Pre	34.6	±	3.9	0.48	33.3	±	5.3	0.30	0.823	0.233	< 0.001
	Post	36.4	±	3.6		34.9	±	5.4				
Assembly task	Pre	22.5	±	3.9	0.81	22.8	±	5.1	0.17	0.006	0.332	< 0.001
	Post	26.4	±	5.6*		23.7	±	5.7				

^*^Significant differences between pre- and post-test

Cohen’s *d*:0.2 = small; *d*:0.5 = moderate; *d*:0.8 = large

*SD *Standard deviation; pre, pre-test; post, post-test

### Manual dexterity

The assembly task showed significant effects of time (*p* < 0.001) and interaction (*p* = 0.006). The post hoc test showed significant improvement after training in the intervention group (22.5 ± 3.9 to 26.4 ± 5.6 counts; *p* < 0.001). The effect size of manual dexterity variables in the intervention group (*d* = 0.32–0.81) was higher than that in the control group (*d* = 0.17–0.36) (Table 2).

### Changes in performance for manual dexterity training mode in the intervention group

**Fig. 2:**
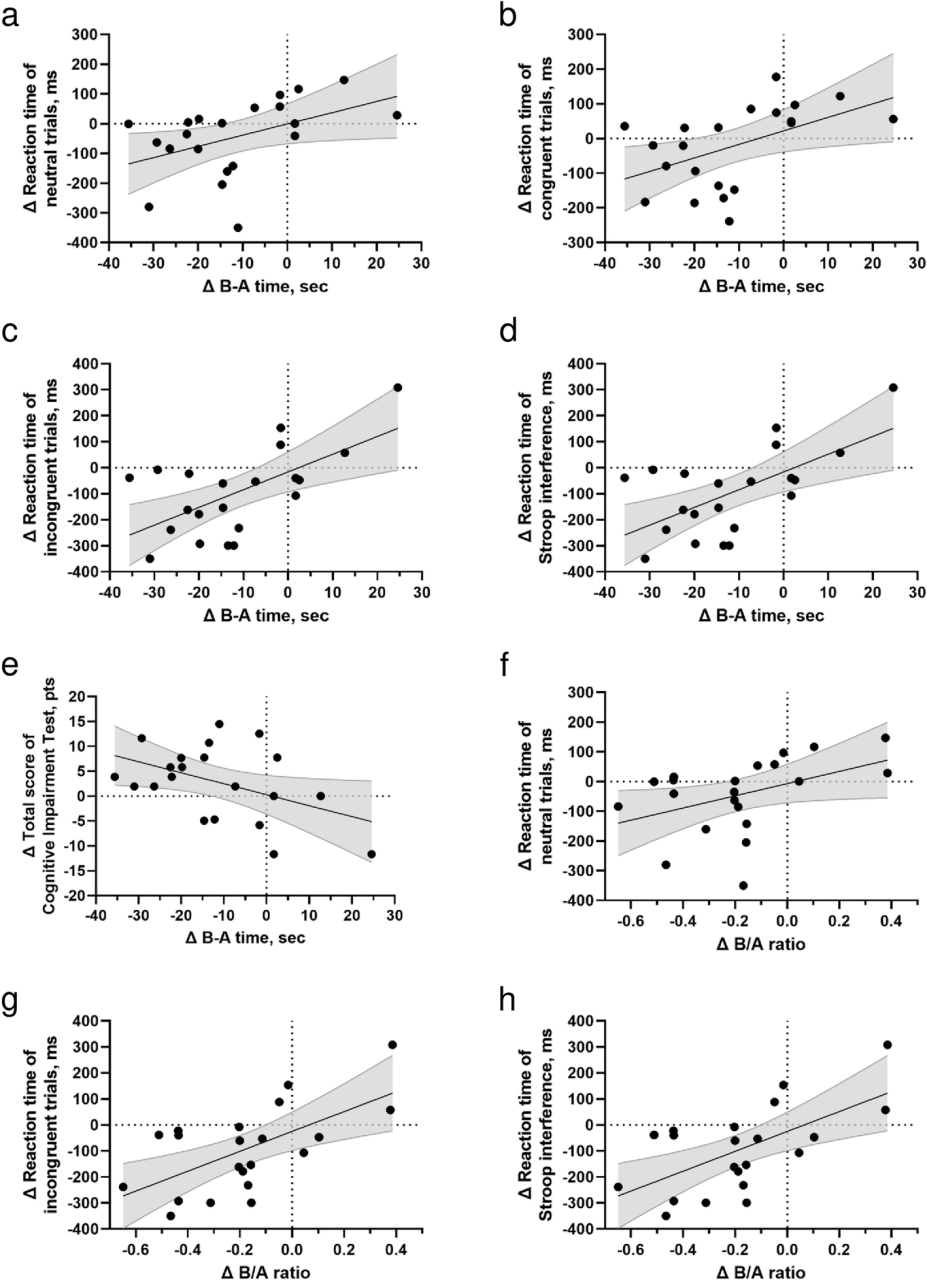
Correlation between training-induced primary outcomes ([post-test] – [pre-test]) and changes in executive variables. Stroop interference calculated as ([incongruent – congruent] of post-test session) – ([incongruent – congruent] of pre-test session); B-A time calculated as ([difference time of B- and A-modes] of last-week intervention period) – ([difference time of B- and A-modes] of first-time intervention period); B/A ratio calculated as ([ratio of B- and A-modes] of last-time intervention period) – ([ratio of B- and A-modes] of first-time intervention period)

In the intervention group, improved B-A time was positively related to the reaction time of neutral (*r* = 0.449, *p* = 0.041), congruent (*r* = 0.497, *p* = 0.022), and incongruent trials (*r* = 0.621, *p* = 0.003), Stroop interference (*r* = 0.436, *p* = 0.048), and the total score of the Cognitive Impairment Test (*r* = 0.450, *p* = 0.041) (Fig. 2a–e).

The B/A ratio was favorably related to the reaction time of neutral (*r* = 0.434, *p* = 0.049) and incongruent trials (*r* = 0.620, *p* = 0.003) and Stroop interference (*r* = 0.511, *p* = 0.018) (Fig. 2f–h).

## Discussion

The present study is one of the first intervention studies to examine the effects of home-based manual dexterity training (i.e., grasping and moving an object with visual information) using a digital trail-making peg test device on cognitive function and manual dexterity among older adults. Compared to a previous study using manual dexterity training [12], the present intervention did not significantly enhance simple manual dexterity (number of pegs on right, left, and both hands) in the intervention group, although a moderate effect size for the performance changes of both hands (Cohen’s *d* = 0.57) was observed. In contrast, the performance in the complex manual dexterity task (e.g., assembly task) improved in the intervention group (Table 2). Visual guidance of manual dexterity involves more widespread brain network than that of simple hand movements [1]. Specifically, the grasping, peg movement, and peg insertion tasks performed in this study, all of which required attention to switch between elements, help to activate not only the primary motor area but also the pathways of the primary visual cortex for the parietal reach region, dorsal premotor area, anterior interparietal area, and ventral premotor area [1, 47]. Considering younger adults, an acute experimental study investigating the effects of the different TMT conditions on activation patterns of the PFC demonstrated that, compared with the control condition, both TMT-A and TMT-B conditions are associated with higher activation of the PFC; however, no between-condition differences between TMT-A and TMT-B were observed [18]. In contrast, the results of the present study suggest that task-related activation differences of the PFC between different conditions of the used manual dexterity tasks exist (see Supplementary Table S1 and Fig. 1). Practically, manual dexterity tasks that involve grasping, peg movement, and peg insertion may result in greater stimulation of the PFC than the TMT.

Another experimental study revealed that manual dexterity training with visual neurofeedback activates the anterior PFC more than training with visual neurofeedback [13]. We confirmed that the manual dexterity movement prescribed in this study cumulatively increased the cognitive load on the PFC in the P-, A-, and B-modes (Supplementary Table S1 and Fig. 1). This result supported the increased cognitive load and activation in the PFC [48].

We also found a moderate correlation between improved B-A time and B/A ratio during Stroop task training, which represents executive function (Fig. 2). This study confirms the training effect on manual dexterity over 12 weeks, and this effect, in turn, partially conveys a learning effect regarding all modes (Supplementary Figs. S4 and S5) [49, 50]. However, because the calculation is based on the same point performance during the intervention period, the time difference between B- and A-modes (B-A time) and the ratio of B- and A-modes may indicate not only a learning effect but also an improvement in executive functions [50, 51].

Executive functions include the ability to plan to control goal-directed behavior, directing and maintaining attention, organization, regulation, task switching, and motor control [2]. Our training mode requires the ability to switch from tasks involving the order of numbers and words to finding an answer (peg into a hole), memorization, maintaining attention, and motor control (grasping the peg, identifying a hole, and inserting it into a hole) [28]. These training processes may enhance executive function in older adults. In addition, a separate experimental study demonstrated a high correlation between the TMT-B and Stroop tasks, and that executive function and working memory were mainly associated with speed of rotational hand movements and movement speed in younger adults [4]. The present study also demonstrated a positive relationship with improved manual dexterity and executive function in older adults using manual dexterity training, which have cognitive load.

Except executive function, other cognitive functions (such as those associated with components of the Cognitive Impairment Test) did not show significant improvements following the intervention. The training effect on executive function has been demonstrated in a relatively shorter period (1–3 months) [52] than that on general cognitive function [53]. Since certain evidence in the literature suggests that a longer training duration and higher frequency of training sessions are associated with greater effects on measures of global cognitive function among older adults [53], our relatively short training duration might not be sufficient for measurable changes in global cognitive performance. Thus, it is possible that long-term training will improve general cognitive function among older adults. One previously mentioned limitation was that the intervention period was short. There are certain limitations that should be considered when interpreting the study findings. First, we did not perform follow-up assessments and thus could not derive conclusions regarding the time course of intervention-related effects (e.g., reversibility of the effects, delayed effects). Second, our fNIRS systems allow only the quantification of cortical activity changes within the PFC. Moreover, we have used only a low-pass filter based on a similar previous study [18]; thus, future studies should use variable filters to remove systemic physiological artifacts as recommended by the fNIRS guidelines [54]. Since a previous study using functional magnetic resonance imaging (fMRI) noticed that several areas in the human brain exhibited changes in the activation patterns following hand movement or dexterity training [12], we recommend that future studies should assess and compare the pre- and post-training activation patterns in other relevant brain areas (e.g., primary motor area, primary visual cortex for the parietal reach region, dorsal premotor area, anterior interparietal area, and ventral premotor area). Third, in the current study, only healthy older adults were included, which limits the generalizability of our findings. Based on our promising results, further research should aim to assess the effects of home-based manual dexterity training in other cohorts (e.g., older adults with mild cognitive impairment). Finally, it was beyond the scope of this study to examine sex differences [10], although we stratified sex [22].

## Conclusions

The findings of the current study suggest that home-based manual dexterity training can improve the performance in a complex manual dexterity task and executive functioning in older adults. Cognizant of the limitations of the present study, future studies should consider sex differences and investigate the effects of manual dexterity on cognitive function and manual dexterity among older adults who are frail and have mild cognitive impairment, dementia, or other impairments.

## Supplementary Information

**Additional file 1: Table S1.** Changes of Oxy-Hb and Deoxy-Hb during each task. **Fig. S1.** Flow diagram from initial contact with participants to study completion. **Fig. S2.** Changes in oxy-Hb level in the frontal lobe over time in each mode. **Fig. S3.** Description of the digital trail-making peg test device. **Fig. S4.** Change in performance training mode of A-, B-, B-A time, and B/A ratio. A lower time for the A- and B-modes indicates a positive performance. A lower B-A time and B/A ratio indicate positive executive function. **Fig. S5. **Change in performance training mode of C-, F-, M-, average P-, and V-mode during the intervention. Lower values of F-, average P-, and V-modes and higher values of C- and M-modes indicate positive performance

## Data Availability

The datasets used and/or analyzed during the current study are available from the corresponding author on reasonable request.

## Abbreviations

ANOVA: Analysis of variance
fMRI: Functional magnetic resonance imaging
fNIRS: Functional near-infrared spectroscopy
PFC: Prefrontal cortex
TMT: Trail-making test

## References

[1] Sobinov AR, Bensmaia SJ (2021). The neural mechanisms of manual dexterity. Nat Rev Neurosci.

[2] Kobayashi-Cuya KE, Sakurai R, Sakuma N, Suzuki H, Yasunaga M, Ogawa S (2018). Hand dexterity, not handgrip strength, is associated with executive function in Japanese community-dwelling older adults: a cross-sectional study. BMC Geriatr.

[3] Seer C, Adab HZ, Sidlauskaite J, Dhollander T, Chalavi S, Gooijers J (2022). Bridging cognition and action: executive functioning mediates the relationship between white matter fiber density and complex motor abilities in older adults. Aging (Albany NY).

[4] Rodríguez-Aranda C, Mittner M, Vasylenko O (2016). Association between executive functions, working memory, and manual dexterity in young and healthy older adults: an exploratory study. Percept Mot Skills.

[5] Carmeli E, Patish H, Coleman R (2003). The aging hand. J Gerontol A Biol Sci Med Sci.

[6] Ranganathan VK, Siemionow V, Sahgal V, Yue GH (2001). Effects of aging on hand function. J Am Geriatr Soc.

[7] Diamond A (2013). Executive functions. Annu Rev Psychol.

[8] Carment L, Abdellatif A, Lafuente-Lafuente C, Pariel S, Maier MA, Belmin J (2018). Manual dexterity and aging: a pilot study disentangling sensorimotor from cognitive decline. Front Neurol.

[9] Firth J, Firth JA, Stubbs B, Vancampfort D, Schuch FB, Hallgren M (2018). Association between muscular strength and cognition in people with major depression or bipolar disorder and healthy controls. JAMA Psychiat.

[10] Vasylenko O, Gorecka MM, Rodríguez-Aranda C. Manual dexterity in young and healthy older adults. 2. Association with cognitive abilities. Dev Psychobiol. 2018;60:428–39. 10.1002/dev.21618 29498421

[11] Corti EJ, Johnson AR, Riddle H, Gasson N, Kane R, Loftus AM (2017). The relationship between executive function and fine motor control in young and older adults. Hum Mov Sci.

[12] Naito E, Morita T, Hirose S, Kimura N, Okamoto H, Kamimukai C (2021). Bimanual digit training improves right-hand dexterity in older adults by reactivating declined ipsilateral motor-cortical inhibition. Sci Rep.

[13] Ota Y, Takamoto K, Urakawa S, Nishimaru H, Matsumoto J, Takamura Y (2020). Motor imagery training with neurofeedback from the frontal pole facilitated sensorimotor cortical activity and improved hand dexterity. Front Neurosci.

[14] Vasylenko O, Gorecka MM, Waterloo K, Rodríguez-Aranda C (2022). Reduction in manual asymmetry and decline in fine manual dexterity in right-handed older adults with mild cognitive impairment. Laterality.

[15] de Paula JJ, Albuquerque MR, Lage GM, Bicalho MA, Romano-Silva MA, Malloy-Diniz LF (2016). Impairment of fine motor dexterity in mild cognitive impairment and Alzheimer's disease dementia: association with activities of daily living. Braz J Psychiatry.

[16] Ranganathan VK, Siemionow V, Sahgal V, Liu JZ, Yue GH (2001). Skilled finger movement exercise improves hand function. J Gerontol A Biol Sci Med Sci.

[17] Abe T, Fujii K, Hyodo K, Kitano N, Okura T (2018). Effects of acute exercise in the sitting position on executive function evaluated by the Stroop task in healthy older adults. J Phys Ther Sci.

[18] Lancia S, Choi J, Baek J, Mammarella S, Bianco D, Quaresima V (2018). Trail making test induces prefrontal cortex activation as revealed by a cw wearable-wireless fNIRS/DOT imager. Adv Exp Med Biol.

[19] Bakhshipour E, Koiler R, Milla K, Getchell N, Ayaz H, Asgher U (2021). Understanding the Cognitive Demands of the Purdue Pegboard Test: An fNIRs Study. Advances in Neuroergonomics and Cognitive Engineering.

[20] Kaufer DI, Williams CS, Braaten AJ, Gill K, Zimmerman S, Sloane PD (2008). Cognitive screening for dementia and mild cognitive impairment in assisted living: comparison of 3 tests. J Am Med Dir Assoc.

[21] Lakens D, Caldwell A (2019). Simulation-based power-analysis for factorial ANOVA designs. PsyArXiv.

[22] Roivainen E, Suokas F, Saari A (2021). An examination of factors that may contribute to gender differences in psychomotor processing speed. BMC Psychol.

[23] Granström F, Hedlund M, Lindström B, Eriksson S (2019). Test-retest reliability of the twenty-five-hole peg test in patients who had a stroke. BMJ Open.

[24] Wagner S, Helmreich I, Dahmen N, Lieb K, Tadic A (2011). Reliability of three alternate forms of the trail making tests a and B. Arch Clin Neuropsychol.

[25] Inoue T, Nagata K, Tateoka K, Seol J, Yoon J, Tsuji T (2022). Relationship between performance on the Digital Trail Making Peg test and cognitive function in older adults. Nihon Ronen Igakkai Zasshi.

[26] Hagenaars SP, Cox SR, Hill WD, Davies G, Liewald DCM; CHARGE consortium Cognitive Working Group, et al. Genetic contributions to Trail Making Test performance in UK Biobank. Mol Psychiatry. 2018;23:1575–83. 10.1038/mp.2017.189 28924184

[27] Lee T, Mosing MA, Henry JD, Trollor JN, Ames D, Martin NG (2012). Genetic influences on four measures of executive functions and their covariation with general cognitive ability: the Older Australian Twins Study. Behav Genet.

[28] Abe T, Jindo T, Soma Y, Tsunoda K, Kitano N, Yoon JY (2015). Validity and reliability of the "Trail Making Peg" test as a performance measurement for evaluating the cognitive function. Nihon Ronen Igakkai Zasshi.

[29] Huppert TJ (2016). Commentary on the statistical properties of noise and its implication on general linear models in functional near-infrared spectroscopy. Neurophotonics.

[30] Yücel MA, Lühmann AV, Scholkmann F, Gervain J, Dan I, Ayaz H (2021). Best practices for fNIRS publications. Neurophotonics.

[31] Klem GH, Lüders HO, Jasper HH, Elger C. The ten-twenty electrode system of the International Federation. The International Federation of Clinical Neurophysiology. Electroencephalogr Clin Neurophysiol Suppl. 1999;52:3–6. 10590970

[32] Virtanen J, Noponen T, Kotilahti K, Virtanen J, Ilmoniemi RJ (2011). Accelerometer-based method for correcting signal baseline changes caused by motion artifacts in medical near-infrared spectroscopy. J Biomed Opt.

[33] Hitoshi T, Aibara K. Measurement and Evaluation of brain activity for train drivers using wearable NIRS. Neuroimaging-neurobiology, multimodal and network applications. IntechOpen. 2019; 10.5772/intechopen.90499

[34] Raz A, Shapiro T, Fan J, Posner MI (2002). Hypnotic suggestion and the modulation of Stroop interference. Arch Gen Psychiatry.

[35] Byun K, Hyodo K, Suwabe K, Ochi G, Sakairi Y, Kato M (2014). Positive effect of acute mild exercise on executive function via arousal-related prefrontal activations: an fNIRS study. Neuroimage.

[36] Laird AR, McMillan KM, Lancaster JL, Kochunov P, Turkeltaub PE, Pardo JV (2005). A comparison of label-based review and ALE meta-analysis in the Stroop task. Hum Brain Mapp.

[37] Ichikawa M, Inada H, Nakahara S (2020). Effect of a cognitive test at license renewal for older drivers on their crash risk in Japan. Inj Prev.

[38] National Police Agency, Japan. Cognitive impairment screening test for senior drivers. https://www.npa.go.jp/english/bureau/traffic/index.html. Accessed 10 Oct 2022.

[39] Tiffin J, Asher EJ (1948). The Purdue pegboard; norms and studies of reliability and validity. J Appl Psychol.

[40] Sheikh JI, Yesavage JA (1986). Geriatric Depression Scale (GDS) recent evidence and development of a shorter version. Clin Gerontol.

[41] Washburn RA, Smith KW, Jette AM, Janney CA (1993). The Physical Activity Scale for the Elderly (PASE): development and evaluation. J Clin Epidemiol.

[42] Lubben J, Blozik E, Gillmann G, Iliffe S, von Renteln KW, Beck JC (2006). Performance of an abbreviated version of the Lubben Social Network Scale among three European community-dwelling older adult populations. Gerontologist.

[43] Buysse DJ, Hall ML, Strollo PJ, Kamarck TW, Owens J, Lee L (2008). Relationships between the Pittsburgh Sleep Quality Index (PSQI), Epworth Sleepiness Scale (ESS), and clinical/polysomnographic measures in a community sample. J Clin Sleep Med.

[44] Phillips A, Haudiquet V (2003). ICH E9 guideline 'Statistical principles for clinical trials': a case study. Stat Med.

[45] Zhu W. p < 0.05, < 0.01, < 0.001, < 0.0001, < 0.00001, < 0.000001, or < 0.0000001 …. J Sport Health Sci. 2016;5:77–9. 10.1016/j.jshs.2016.01.019 PMC6191982 30356881

[46] Cohen J. Statistical power analysis for the behavioral sciences. Routledge Academic; 1998.

[47] Logue SF, Gould TJ (2014). The neural and genetic basis of executive function: attention, cognitive flexibility, and response inhibition. Pharmacol Biochem Behav.

[48] Han W, Gao L, Wu J, Pelowski M, Liu T (2019). Assessing the brain 'on the line': An ecologically-valid assessment of the impact of repetitive assembly line work on hemodynamic response and fine motor control using fNIRS. Brain Cogn.

[49] Buck KK, Atkinson TM, Ryan JP (2008). Evidence of practice effects in variants of the Trail Making Test during serial assessment. J Clin Exp Neuropsychol.

[50] Mckeown K, Richards E, Richardson J, Tales A. The trails making test. does a single trial reflect performance capability? OBM Neurobiol. 2021;5:100;

[51] Martin TA, Hoffman NM, Donders J (2003). Clinical utility of the trail making test ratio score. Appl Neuropsychol.

[52] Chen FT, Etnier JL, Chan KH, Chiu PK, Hung TM, Chang YK (2020). Effects of exercise training interventions on executive function in older adults: a systematic review and meta-analysis. Sports Med.

[53] Wollesen B, Wildbredt A, van Schooten KS, Lim ML, Delbaere K (2020). The effects of cognitive-motor training interventions on executive functions in older people: a systematic review and meta-analysis. Eur Rev Aging Phys Act.

[54] Yücel MA,Lühmann Av, Scholkmann F, Gervain J, Dan I, Ayaz H,  (2021). Best practices for fNIRS publications Neurophoton.

