# Supplementary material for: Effects of home-based manual dexterity training on cognitive function among older adults: a randomized controlled trial
Source: Eur Rev Aging Phys Act. 2023 Apr 22;20:9. doi: 10.1186/s11556-023-00319-2 (PMC10121426; doi:10.1186/s11556-023-00319-2)
Supplement: Supplementary file 1 — Additional file 1: Table S1. Changes of Oxy-Hb and Deoxy-Hb during each task. Fig. S1. Flow diagram from initial contact with participants to study completion. Fig. S2. Changes in oxy-Hb level in the frontal lobe over time in each mode. Fig. S3. Description of the digital trail-making peg test device. Fig. S4. Change in performance training mode of A-, B-, B-A time, and B/A ratio. A lower time for the A- and B-modes indicates a positive performance. A lower B-A time and B/A ratio indicate positive executive function. Fig. S5. Change in performance training mode of C-, F-, M-, average P-, and V-mode during the intervention. Lower values of F-, average P-, and V-modes and higher values of C- and M-modes indicate positive performance [file 11556_2023_319_MOESM1_ESM.pdf]

Supplementary Material

Effects of home-based manual dexterity training on cognitive function among older adults: a randomized controlled trial

Jaehoon Seol, Namhoon Lim, Koki Nagata, and Tomohiro Okura

Correspondence should be addressed to Jaehoon Seol, PhD. Email: seol.jaehoon.ge@u.tsukuba.ac.jp

This PDF file includes:

Methods
Table S1
Figs. S1 to S5
References for SI reference citations

Supplementary Methods

Interventions

A- and B-modes (executive)

A-mode was trail-making peg test part A, whereas B-mode was trail-making peg test part B. The A-mode involved 25 pegs in distal place as well as grasping a peg with one side of the hand and moving it to a proximal hole in the displayed numerical order (1→2→3, …, 24→25). The B-mode involved combined numbers and Japanese characters in "hiragana" order; the participants grasped a peg with one side of their hand and alternately moved the peg to a proximal hole in the displayed order (1→[a]→2→[i]→3→[u], …, [shi]→13). Both A- and B-modes involved measuring the total time until the last peg was inserted. The validity and reliability of trail-making peg test parts A and B (A- and B-modes) for cognitive function were confirmed in older adults [1].

C-mode (maintenance of concentration)

Similar to the A-mode, the participants grasped a peg in the distal hole with one side of the hand and moved to the proximal hole in the displayed numerical order. Subsequently, the inserted peg was moved to the distal hole from the proximal hole in the randomly displayed numerical order once again. This insertion and removal task was repeated for 100 s. The total number of moved pegs for 100 s was recorded.

F-mode (attention)

Five “<” or “>” were marked at the same row next to the proximal hole, and there was only one different direction. The participants were tasked with quickly inserting a peg to a different displayed direction as soon as possible. After the first row was completed, the next row was displayed. The total time required for completing 20 repetitions of this task was recorded.

M-mode (memory)

Five squares were randomly marked next to the proximal hole, and the participants were tasked with memorizing the square sites for 10 s. C-mode was provided as an interference task for 20 s. After the interference task, the participants inserted a peg into the hole they had memorized as the square site. The number of correct sites (score of 0-5) was recorded.

P-mode (hand dexterity)

The P-mode was a peg moving test and consisted of four sub-modes displaying the start location (top of the left or right side) and direction of progress (right/left to left/right or upper to bottom). The participants grasped a peg in the distal hole with one side of their hand and quickly moved the peg to the proximal hole as soon as possible. The total time until the insertion of the last peg was recorded.

V-mode (visuospatial)

One square was randomly marked next to the proximal hole. The participants quickly inserted a peg in the displayed square of the hole as soon as possible.

Subsequently, the square was randomly marked in the next location. The total time required for completing 20 repetitions of this task was recorded.

Table S1 . Changes of Oxy-Hb and Deoxy-Hb during each task

			Baseline	25%	50%	75%	100%	Group *P*	Time *P*	Interaction *P*
Oxy-Hb	Ch 1	P-mode	-0.00014 ± 0.00082	0.00170 ± 0.00082	0.00478 ± 0.00082	0.00530 ± 0.00082	0.00528 ± 0.00082	<0.001	<0.001	<0.001
		A-mode	0.00027 ± 0.00081	0.00249 ± 0.00081	0.00529 ± 0.00081	0.00826 ± 0.00081	0.00935 ± 0.00081			
		B-mode	-0.00008 ± 0.00085	0.00217 ± 0.00086	0.00493 ± 0.00086	0.00825 ± 0.00086	0.01090 ± 0.00086			
	Ch 2	P-mode	0.00000 ± 0.00091	0.00191 ± 0.00091	0.00522 ± 0.00091	0.00552 ± 0.00091	0.00569 ± 0.00091	<0.001	<0.001	<0.001
		A-mode	0.00029 ± 0.00092	0.00245 ± 0.00092	0.00514 ± 0.00092	0.00760 ± 0.00092	0.00925 ± 0.00092			
		B-mode	-0.00035 ± 0.00096	0.00265 ± 0.00097	0.00606 ± 0.00097	0.00977 ± 0.00097	0.01243 ± 0.00097			
	Ch 3	P-mode	0.00015 ± 0.00072	0.00206 ± 0.00072	0.00512 ± 0.00072	0.00577 ± 0.00072	0.00519 ± 0.00072	<0.001	<0.001	<0.001
		A-mode	0.00010 ± 0.00071	0.00262 ± 0.00071	0.00529 ± 0.00071	0.00816 ± 0.00071	0.00905 ± 0.00071			
		B-mode	0.00011 ± 0.00075	0.00272 ± 0.00075	0.00499 ± 0.00075	0.00780 ± 0.00075	0.01001 ± 0.00075			
	Ch 4	P-mode	-0.00006 ± 0.00081	0.00114 ± 0.00081	0.00440 ± 0.00081	0.00462 ± 0.00081	0.00400 ± 0.00081	<0.001	<0.001	<0.001
		A-mode	-0.00001 ± 0.00081	0.00246 ± 0.00081	0.00478 ± 0.00081	0.00827 ± 0.00081	0.00951 ± 0.00081			
		B-mode	-0.00005 ± 0.00084	0.00194 ± 0.00085	0.00464 ± 0.00085	0.00803 ± 0.00085	0.01036 ± 0.00085			
Deoxy-Hb	Ch 1	P-mode	0.00001 ± 0.00047	-0.00078 ± 0.00047	-0.00145 ± 0.00047	-0.00181 ± 0.00047	-0.00221 ± 0.00047	0.096	<0.001	0.873
		A-mode	0.00009 ± 0.00046	-0.00106 ± 0.00046	-0.00177 ± 0.00046	-0.00311 ± 0.00046	-0.00323 ± 0.00046			
		B-mode	-0.00006 ± 0.00050	-0.00100 ± 0.00050	-0.00187 ± 0.00050	-0.00247 ± 0.00050	-0.00281 ± 0.00050			
	Ch 2	P-mode	0.00001 ± 0.00054	-0.00005 ± 0.00054	-0.00060 ± 0.00054	-0.00133 ± 0.00054	-0.00147 ± 0.00054	0.018	<0.001	0.992
		A-mode	0.00025 ± 0.00054	-0.00020 ± 0.00054	-0.00092 ± 0.00054	-0.00100 ± 0.00054	-0.00139 ± 0.00054			
		B-mode	-0.00034 ± 0.00057	-0.00061 ± 0.00057	-0.00146 ± 0.00057	-0.00195 ± 0.00057	-0.00238 ± 0.00057			
	Ch 3	P-mode	-0.00007 ± 0.00036	-0.00041 ± 0.00036	-0.00176 ± 0.00036	-0.00280 ± 0.00036	-0.00303 ± 0.00036	0.003	<0.001	0.717
		A-mode	-0.00005 ± 0.00036	-0.00165 ± 0.00036	-0.00237 ± 0.00036	-0.00320 ± 0.00036	-0.00368 ± 0.00036			
		B-mode	-0.00003 ± 0.00038	-0.00144 ± 0.00038	-0.00259 ± 0.00038	-0.00351 ± 0.00038	-0.00385 ± 0.00038			
	Ch 4	P-mode	0.00000 ± 0.00041	-0.00038 ± 0.00041	-0.00189 ± 0.00041	-0.00315 ± 0.00041	-0.00341 ± 0.00041	0.320	<0.001	0.863
		A-mode	0.00000 ± 0.00041	-0.00116 ± 0.00041	-0.00233 ± 0.00041	-0.00307 ± 0.00041	-0.00381 ± 0.00041			
		B-mode	-0.00008 ± 0.00043	-0.00119 ± 0.00043	-0.00227 ± 0.00043	-0.00289 ± 0.00043	-0.00344 ± 0.00043			

Data are shown as the Mean ± SE.

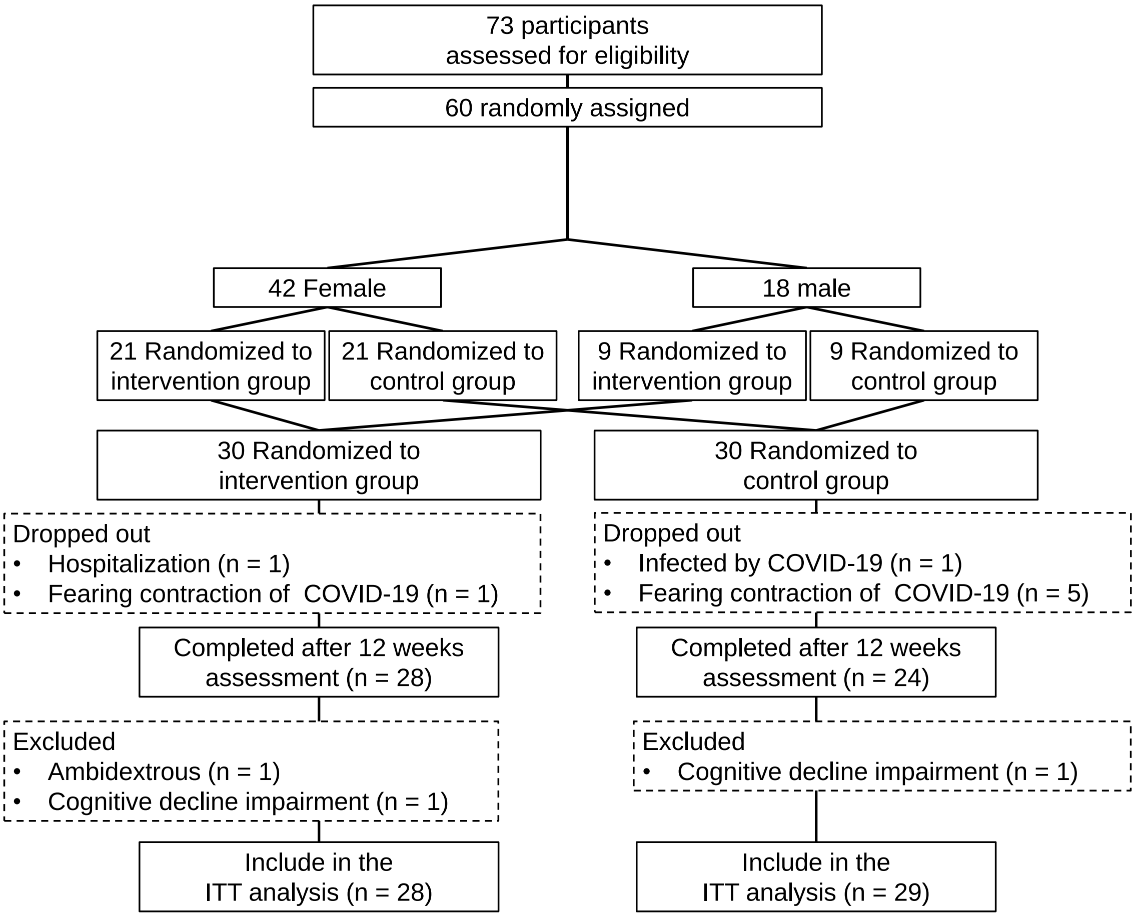

Fig. S1 Flow diagram from initial contact with participants to study completion

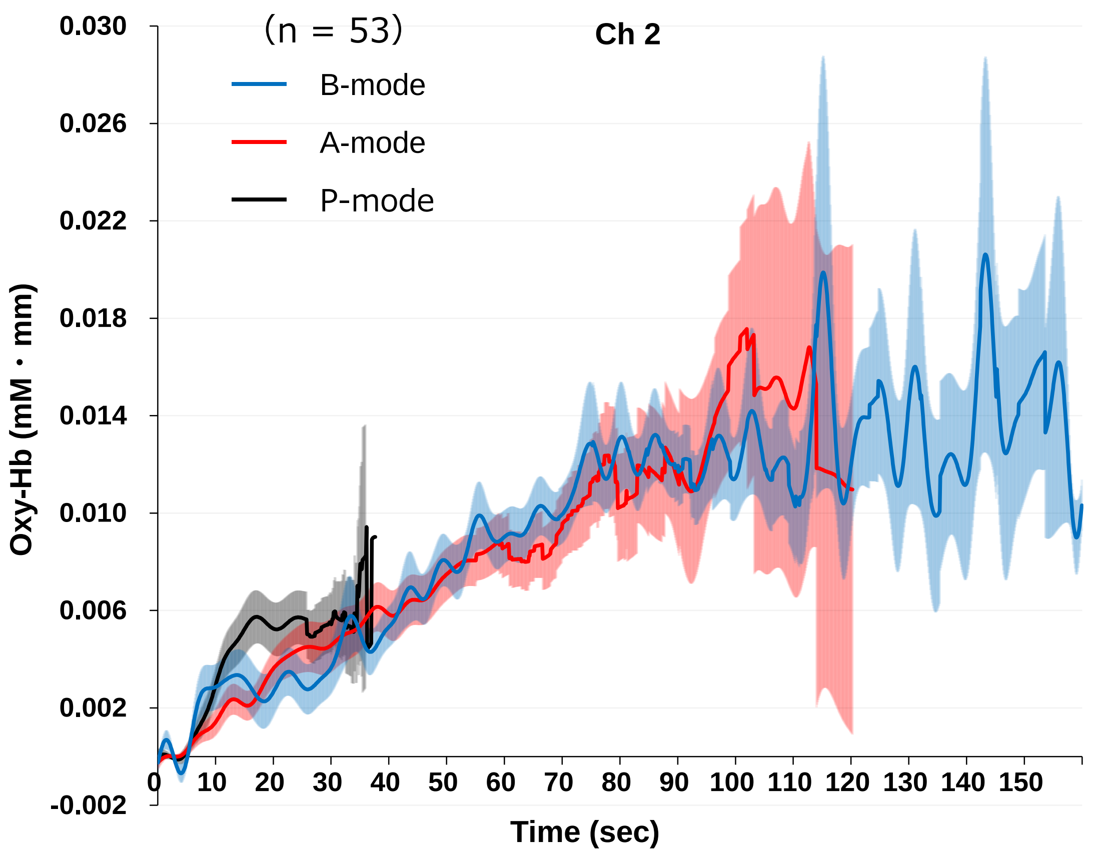

Fig. S2 Changes in oxy-Hb level in the frontal lobe over time in each mode

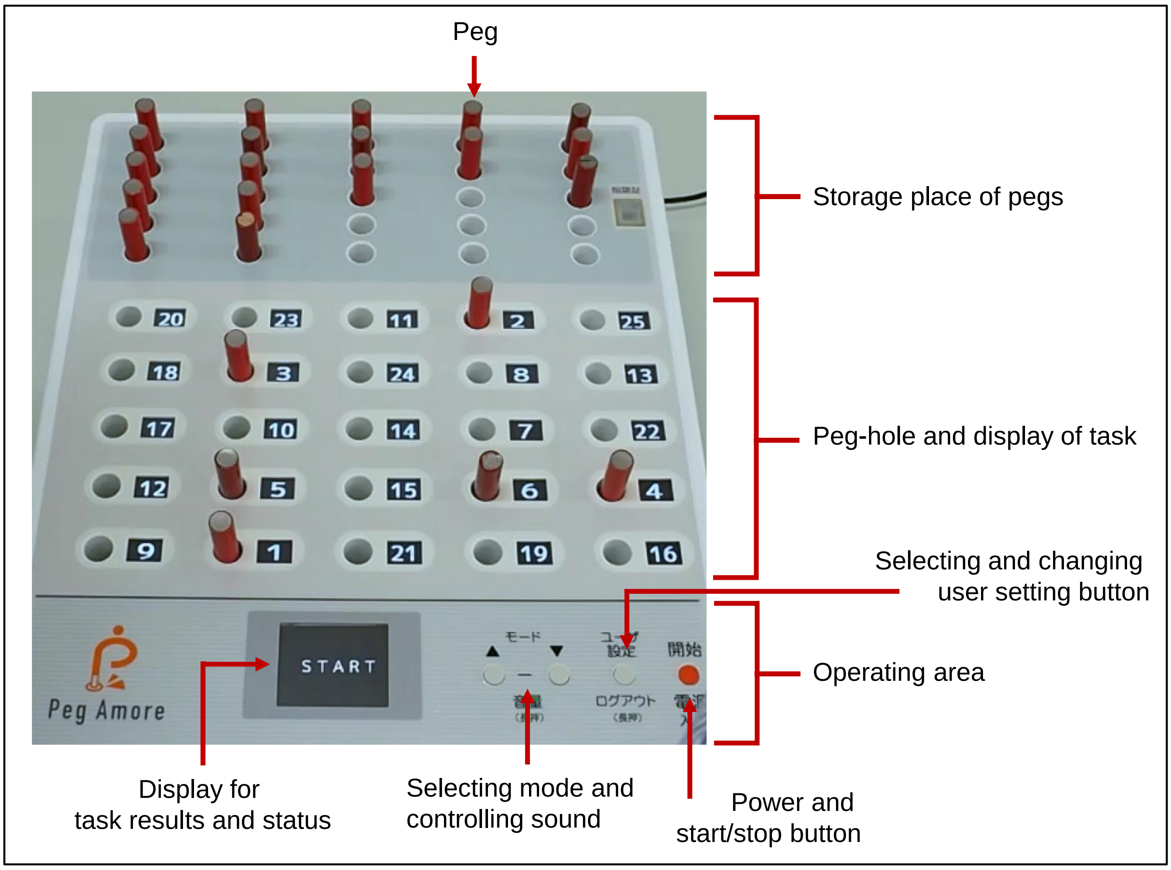

Fig. S3 Description of the digital trail-making peg test device

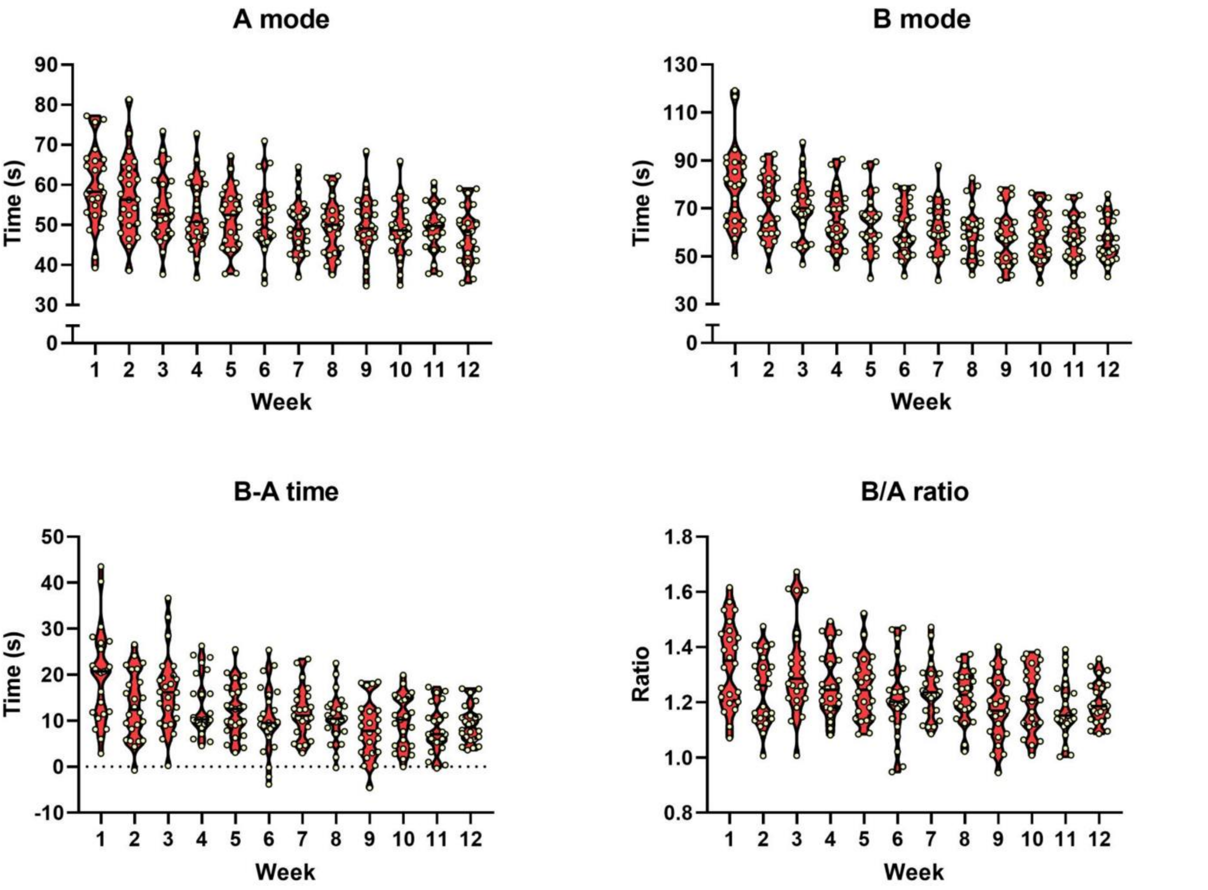

Fig. S4 Change in performance training mode of A-, B-, B-A time, and B/A ratio.

A lower time for the A- and B-modes indicates a positive performance. A lower B-A time and B/A ratio indicate positive executive function

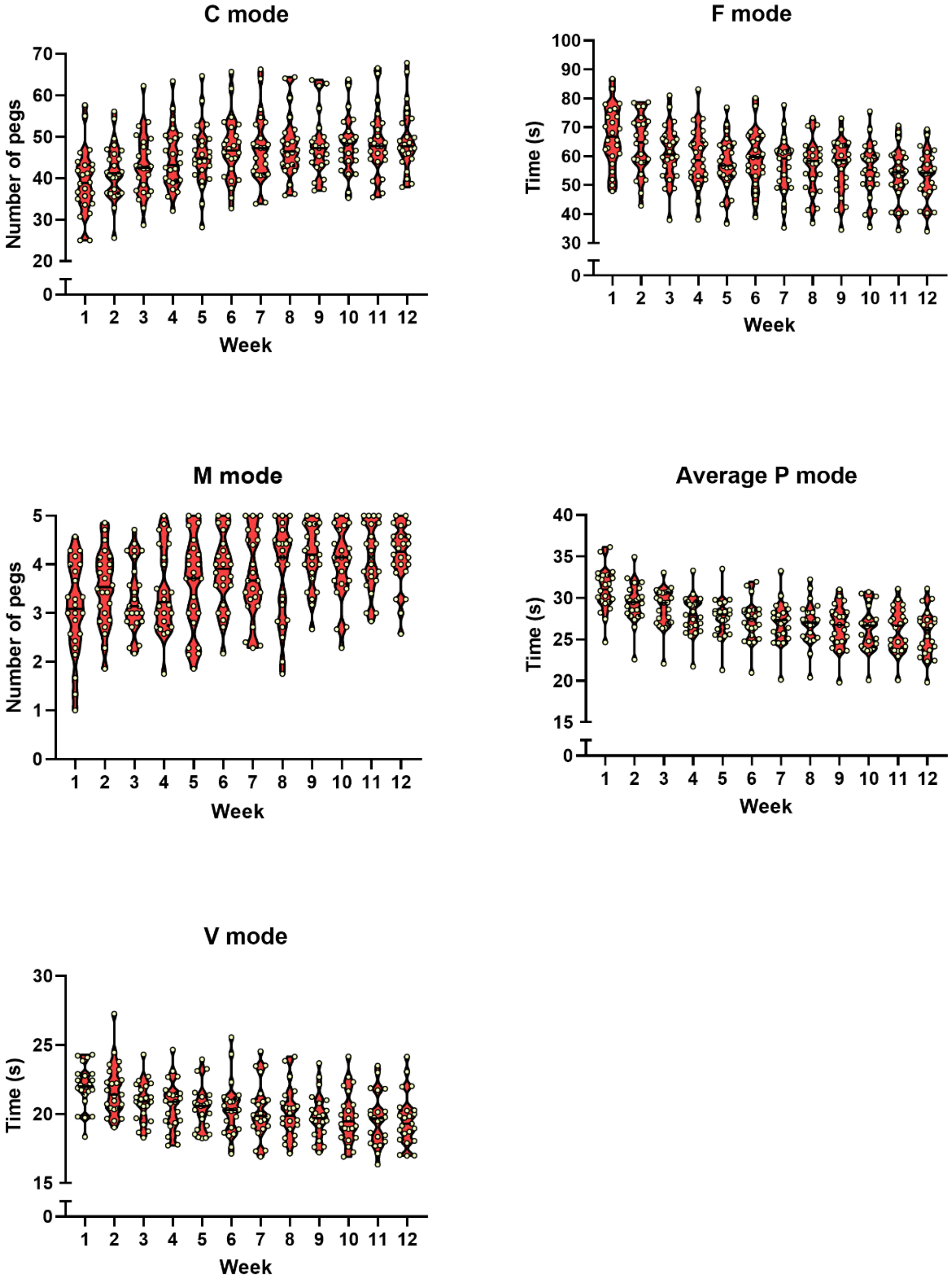

Fig. S5 Change in performance training mode of C-, F-, M-, average P-, and V-mode during the intervention.

Lower values of F-, average P-, and V-modes and higher values of C- and M-modes indicate positive performance

References

1. Abe T, Jindo T, Soma Y, Tsunoda K, Kitano N, Yoon JY, et al. Validity and reliability of the "Trail Making Peg" test as a performance measurement for evaluating the cognitive function. Nihon Ronen Igakkai Zasshi. 2015;52:71–78. doi:10.3143/geriatrics.52.71
